# Predicting postoperative pancreatic fistula after robotic pancreatoduodenectomy using International Study Group on Pancreatic Surgery and fistula risk scores: European multicentre retrospective cohort study

**DOI:** 10.1093/bjsopen/zraf036

**Published:** 2025-05-07

**Authors:** Anouk M L H Emmen, Mahsoem Ali, Bas Groot Koerkamp, Ugo Boggi, I Quintus Molenaar, Olivier R Busch, Thilo Hackert, Luca Moraldi, J Sven Mieog, Daan J Lips, Olivier Saint-Marc, Misha D P Luyer, Susan van Dieren, Geert Kazemier, Felix Nickel, Sebastiaan Festen, Hjalmar C van Santvoort, Emanuele F Kauffmann, Roeland F de Wilde, Mohammad Abu Hilal, Marc G Besselink

**Affiliations:** Department of Surgery, Amsterdam UMC, Location University of Amsterdam, Amsterdam, The Netherlands; Cancer Centre Amsterdam, Amsterdam, The Netherlands; Cancer Centre Amsterdam, Amsterdam, The Netherlands; Department of Surgery, Amsterdam UMC, Location Vrije Universiteit, Amsterdam, The Netherlands; Department of Surgery, Erasmus MC Cancer Institute, Rotterdam, The Netherlands; Division of General and Transplant Surgery, University of Pisa, Pisa, Italy; Department of Surgery, Regional Academic Cancer Centre Utrecht, St. Antonius Hospital and University Medical Centre, Utrecht, The Netherlands; Department of Surgery, Amsterdam UMC, Location University of Amsterdam, Amsterdam, The Netherlands; Cancer Centre Amsterdam, Amsterdam, The Netherlands; Dept. of General, Visceral and Thoracic Surgery, University Hospital Hamburg-Eppendorf, Hamburg, Germany; Department of Oncology and Robotic Surgery, Careggi University Hospital, Florence, Italy; Department of Surgery, Leiden Universitair Medisch Centrum, Leiden, The Netherlands; Department of Surgery, Medisch Spectrum Twente, Enschede, The Netherlands; Service de Chirurgie Digestive, Endocrinienne et Thoracique, Centre Hospitalier Universitaire Orleans, Orleans, France; Department of Surgery, Catharina Hospital, Eindhoven, The Netherlands; Department of Surgery, Amsterdam UMC, Location University of Amsterdam, Amsterdam, The Netherlands; Cancer Centre Amsterdam, Amsterdam, The Netherlands; Department of Surgery, Amsterdam UMC, Location Vrije Universiteit, Amsterdam, The Netherlands; Dept. of General, Visceral and Thoracic Surgery, University Hospital Hamburg-Eppendorf, Hamburg, Germany; Department of Surgery, University Hospital of Heidelberg, Heidelberg, Germany; Department of Surgery, OLVG, Amsterdam, The Netherlands; Department of Surgery, Regional Academic Cancer Centre Utrecht, St. Antonius Hospital and University Medical Centre, Utrecht, The Netherlands; Division of General and Transplant Surgery, University of Pisa, Pisa, Italy; Department of Surgery, Erasmus MC Cancer Institute, Rotterdam, The Netherlands; Department of Surgery, School of Medicine, University of Jordan, Amman, Jordan; Department of Surgery, Southampton University, Southampton, UK; Department of Surgery, Amsterdam UMC, Location University of Amsterdam, Amsterdam, The Netherlands; Cancer Centre Amsterdam, Amsterdam, The Netherlands

## Abstract

**Background:**

Postoperative pancreatic fistula represents the leading cause of morbidity and mortality after robotic pancreatoduodenectomy. Various scores have been proposed to stratify patients based on their postoperative pancreatic fistula risk, including three fistula risk scores, and two International Study Group for Pancreatic Surgery scores. This study compares the performance of these scores in patients undergoing robotic pancreatoduodenectomy.

**Methods:**

This is a multicentre European retrospective study in consecutive patients receiving robotic pancreatoduodenectomy for all indications (April 2014 to December 2021). The performance of the International Study Group for Pancreatic Surgery 4-tier (A–D) risk score, and its 3-tier (A–C) modification (International Study Group for Pancreatic Surgery 3-tier), fistula risk scores, alternative-fistula risk scores and the updated alternative-fistula risk scores in postoperative pancreatic fistula grade B/C prediction were compared based on their discrimination (area under the curve), calibration and clinical utility, evaluated through decision curve analyses.

**Results:**

Overall, 919 patients undergoing robotic pancreatoduodenectomy were included. The rate of grade B/C postoperative pancreatic fistula was 22.2% (*n* = 204). The area under the curve for the five scores differed only slightly: International Study Group for Pancreatic Surgery 0.63 (95% confidence interval (c.i.) 0.58 to 0.67), International Study Group for Pancreatic Surgery 3-tier 0.63 (95% c.i. 0.58 to 0.67), fistula risk scores 0.65 (95% c.i. 0.61 to 0.69), alternative-fistula risk scores 0.64 (95% c.i. 0.60 to 0.68) and updated alternative-fistula risk scores 0.65 (95% c.i. 0.60 to 0.69). The International Study Group for Pancreatic Surgery, International Study Group for Pancreatic Surgery 3-tier, fistula risk scores and alternative-fistula risk scores underestimated the risk of postoperative pancreatic fistula. In contrast, the updated alternative-fistula risk score was well-calibrated at low predicted risks, but overestimated postoperative pancreatic fistula risk for high-risk patients. In decision curve analyses, the updated alternative-fistula risk score showed a higher clinical utility compared with the four other risk scores.

**Conclusion:**

The clinical utility of the updated alternative-fistula risk score for robotic pancreatoduodenectomy slightly outperformed the four other fistula risk scores, and might be used for patient counselling and patient stratification in clinical practice and research.

## Teaser

This is a multicentre international retrospective study in consecutive patients after robotic pancreatoduodenectomy (RPD) for all indications, comparing the ISGPS and FRS risk scores. The clinical utility of the updated alternative-fistula risk score for RPD slightly outperformed the four other fistula risk scores, and might be used for patient counselling and patient stratification in clinical practice.

## Introduction

Pancreatoduodenectomy represents the treatment of choice for neoplasms in the pancreatic head and periampullary region but is associated with considerable morbidity rates^1^. In recent years, robotic pancreatoduodenectomy (RPD) has been gaining popularity^2^ with the aim of reducing the morbidity rate and enhancing postoperative recovery^3,4^. Patients are often selected for RPD based on smaller tumour size and/or the absence of vascular contact, resulting in an overrepresentation of patients with soft pancreatic textures and, consequently, a higher risk of postoperative pancreatic fistula (POPF) in RPD cohorts.

POPF is one of the most feared complications after pancreatoduodenectomy, potentially leading to sepsis, haemorrhage and death^5,6^. In the past decade, different risk scores have been developed to predict the risk of POPF. The original fistula risk score (FRS) was based on pancreatic gland texture, pancreatic duct diameter, intraoperative blood loss and definitive pathology^7^. The alternative FRS (a-FRS) was subsequently proposed, including pancreatic texture, duct diameter and body mass index (BMI), followed by the updated alternative-FRS (ua-FRS) including gland texture, pancreatic duct diameter, BMI and sex, and was specifically validated for both minimally invasive and open pancreatoduodenectomy^8,9^.

In 2021, the International Study Group for Pancreatic Surgery (ISGPS) published a simple 4-tier (that is A–D) risk classification for POPF based on two risk factors: pancreatic gland texture and main pancreatic duct size^10^. A 2023 validation of the ISGPS classification for open and minimally invasive pancreatoduodenectomy (MIPD) using data from the nationwide Dutch Pancreatic Cancer Audit reported similar performance with a simplified 3-tier ISGPS risk classification (that is A–C), which was also associated with a more balanced patient distribution^11^.

These new ISGPS scores have neither been specifically validated in patients undergoing RPD nor head-to-head compared with the three FRS^8,10,11^. Therefore, this study aimed to validate the ISGPS 4-tier and ISGPS 3-tier scores and to compare their predictive performance with the FRS, a-FRS and ua-FRS in a large European cohort of patients undergoing RPD.

## Methods

This international multicentre retrospective cohort study included consecutive patients receiving RPD for all indications from the first procedure in ten centres from four European countries within the European consortium of Minimally Invasive Pancreatic Surgery (E-MIPS) (April 2014 to December 2021). Data were retrieved from the E-MIPS registry and additional data were added by the local study coordinator in each centre. Follow-up data were collected during hospital stay and, in the case of discharge, within 30 days. All data were anonymized. Ethical approval was waived by the Ethical Committee of the Amsterdam UMC.

### Data collection

Patient characteristics included age, sex, BMI, American Society of Anesthesiologists (ASA) score and preoperative pathology. Intraoperative variables included gland texture (assessed intraoperatively by the surgeon), pancreatic duct size (measured intraoperatively at the transection point of the pancreas), operating time, intraoperative blood loss and conversion. Postoperative outcomes included major morbidity rate (Clavien–Dindo ≥III)^12^, POPF grade B/C^13^, postpancreatectomy haemorrhage (PPH, grade B/C)^14^, bile leakage (grade B/C)^15^, delayed gastric emptying (DGE, grade B/C)^16^, chyle leakage (grade B/C)^17^, reoperation on, readmission, in-hospital/30-day mortality rate, pancreatic ductal adenocarcinoma (PDAC) and malignant pathology (adenocarcinoma).

### Risk scores

Risk for POPF was calculated according to the ISGPS risk classification (using gland texture and pancreatic duct size), the ISGPS 3-tier risk classification (using gland texture and pancreatic duct size), the FRS risk classification (using gland texture, pathology, pancreatic duct size and intraoperative blood loss), the a-FRS risk classification (using gland texture, BMI and pancreatic duct size) and the ua-FRS risk classification (using pancreatic gland texture, pancreatic duct size, BMI in kg/m^2^ and sex) for each patient^8,10,11^. Calculated percentages of the FRS, a-FRS and ua-FRS formulas were crosschecked using pancreascalculator.com and pancreasclub.com. The ua-FRS risk score was stratified into three risk categories: low risk (<5%), intermediate risk (5–20%) and high risk (>20%)^9^.

### Statistical analysis

Continuous baseline characteristics are presented as median (interquartile range (i.q.r.)) and compared (patients with POPF *versus* patients without POPF) using the Mann–Whitney *U* test. Categorical data are presented as frequencies and percentages and compared using Pearson’s chi-square test.

In accordance with the TRIPOD (Transparent Reporting of a multivariable prediction model for Individual Prognosis Or Diagnosis) statement, the five ISGPS and FRS were compared based on their discrimination, calibration and potential clinical utility^18^. Discrimination was assessed using the area under the receiver operating characteristic curve (AUC). Briefly, the AUC ranges between 0.5 (no discrimination) and 1.0 (perfect discrimination), with higher values indicating that the model can better distinguish between low-risk and high-risk patients. Notably, labelling systems for the AUC are arbitrary, and models with ‘low’ AUC values can have clinical utility, depending on the clinical context and therapeutic consequences. As such, in line with recent statistical recommendations, AUC values are reported without labels, and other performance measures (that is calibration and net benefit) are used to quantify potential clinical utility^19^. Calibration refers to the agreement between the predicted *versus* observed probability of POPF, and was examined using smoothed calibration curves (based on locally estimated scatterplot smoothing). A model with perfect calibration has an intercept of 0 and a slope of 1, that is a perfect diagonal line with predicted probabilities matching exactly with the observed probability of a patient to have POPF. The potential clinical utility of the ISGPS, ISGPS-3, FRS, a-FRS and ua-FRS risk classification models was assessed using decision curve analysis. Briefly, decision curve analysis is a recommended method to examine the clinical consequences of using a risk classification model, which is influenced by both the diagnostic accuracy and calibration of a model^20,21^. In decision curve analysis, the potential clinical utility of a model (quantified in terms of net benefit) can be compared with other models, as well as with a ‘treat all’ and ‘treat none’ strategy. Ideally, a model should show that it has equal or higher net benefit compared with all other strategies. Discrimination, calibration and potential clinical utility were also estimated for all models in a sensitivity analysis, that only included patients from 2018 to 2021, to avoid potential overlap with the development cohort of the ua-FRS score.

Multiple imputation by chained equations was used to account for missing data under the missing at random assumption (*Table S1*). All predictor variables and the outcome (that is presence or absence of POPF) were included in the imputation model to generate ten imputations. Estimates were pooled across imputed data sets using Rubin’s rules. The area under the curve was logit-transformed before pooling, and bootstrapping was used to estimate bias-corrected, accelerated confidence intervals for the difference in AUC between two tests.

All analyses were performed using SPSS v. 28.0.1.1 or R, v. 4.2.1 (R Foundation for Statistical Computing, https://www.r-project.org/foundation/), using the *boot* and *rms* packages. A two-sided *P* value lower than 0.05 was considered statistically significant.

## Results

Overall, 919 patients undergoing RPD were included. The median BMI was 24.8 kg/m^2^ (i.q.r. 22.5–27.5) and 48% of patients were female. Pancreatic texture was most often soft (45.7%), 43% of patients had PDAC (*n* = 396). The overall rate of POPF grade B/C was 22.2% (*n* = 204). The overall reoperation on rate was 11.1% (*n* = 102) with a readmission rate of 17.0% (*n* = 157). The overall in-hospital/30-day mortality rate was 3.7% (*n* = 34). See *Table 1* for more details.

**Table 1:** Baseline characteristics of 919 patients undergoing robotic pancreatoduodenectomy and patients with and without postoperative pancreatic fistula

	RPD (*n* = 919)	POPF grade B/C (*n* = 204)	No POPF (*n* = 715)	*P*
**Baseline characteristics**				
Age (years), median (i.q.r.)	68 (59–74)	69 (61–75)	67 (58–74)	0.059
Sex, male	482 (52.4)	123 (60.3)	358 (50.1)	**0**.**012**
BMI (kg/m^2^), median (i.q.r.)	24.8 (22.5–27.5)	25.6 (23.2–28.3)	24.6 (22.5–27.1)	**0**.**005**
ASA ≥III	260 (28.3)	49 (24.0)	210 (29.4)	0.61
**Intraoperative outcomes**				
Pancreatic texture, soft	421 (45.8)	125 (61.3)	295 (41.3)	**<0**.**001**
Pancreatic duct size (mm), median (i.q.r.)	4 (2–5)	3 (2–4)	4 (2–6)	**<0**.**001**
≤1	110 (12.0)	29 (14.2)	81 (11.3)	
2	140 (15.2)	48 (23.5)	92 (12.9)	
3	153 (16.6)	38 (18.6)	114 (15.9)	
4	118 (12.8)	20 (9.8)	98 (13.7)	
≥5	306 (33.3)	41 (20.1)	263 (36.8)	
Operation time (min), median (i.q.r.)	439 (360–527)	413 (351–508)	440 (363–538)	**0**.**043**
Intraoperative blood loss (ml), median (i.q.r.)	200 (100–400)	200 (100–425)	200 (100–400)	0.56
Conversion	62 (6.7)	15 (7.4)	47 (6.6)	0.82
**Postoperative outcomes**				
Major morbidity rate (C–D≥III)	331 (36.0)	171 (83.8)	159 (22.2)	**<0**.**001**
Postoperative pancreatic fistula, grade B/C	204 (22.2)			
Postpancreatectomy haemorrhage grade B/C	82 (8.9)	47 (23.0)	35 (4.9)	**<0**.**001**
Bile leak grade B/C	67 (7.3)	34 (16.7)	32 (4.5)	**<0**.**001**
Delayed gastric emptying grade B/C	143 (15.6)	67 (32.8)	76 (10.6)	**<0**.**001**
Chyle leak grade B/C	27 (2.9)	4 (2.0)	23 (3.2)	0.49
Reoperation	102 (11.1)	45 (22.1)	57 (8.0)	**<0**.**001**
Readmission	157 (17.1)	63 (30.9)	93 (13.0)	**<0**.**001**
In-hospital/30-day mortality rate	34 (3.7)	12 (5.9)	22 (3.1)	0.13
**Postoperative pathology**				
Malignant	540 (58.8)	114 (55.9)	425 (59.4)	0.41
PDAC	435 (47.3)	80 (39.2)	354 (49.5)	
Cholangiocarcinoma	46 (5.0)	18 (8.8)	28 (3.9)	
Ampullary carcinoma	59 (6.4)	16 (7.8)	43 (6.0)	
Benign	382 (41.6)	90 (44.1)	268 (37.5)	0.41
Adenoma	47 (5.1)	13 (6.4)	26 (3.6)	
Neuroendocrine neoplasm	87 (9.5)	25 (12.3)	61 (8.5)	
Intraductal papillary mucinous neoplasm	56 (6.1)	14 (6.9)	42 (5.9)	
Mucinous cystic neoplasm	16 (1.7)	4 (2.0)	12 (1.7)	
Solid pseudopapillary neoplasm	11 (1.2)	4 (2.0)	7 (1.0)	
Serous cystadenoma	14 (1.5)	0 (0)	13 (1.8)	
Chronic pancreatitis	116 (12.6)	26 (12.7)	77 (10.8)	
Other	21 (2.3)	4 (2.0)	17 (2.4)	

Values are *n* (%) unless otherwise indicated. i.q.r., interquartile range; ASA, American Society of Anesthesiologists classification; BMI, body mass index; C–D, Clavien–Dindo; PDAC, pancreatic ductal adenocarcinoma; RPD, robot pancreatoduodenectomy; POPF, postoperative pancreatic fistula.

Patients’ distribution across the various risk categories and the corresponding POPF rates are displayed in *Table 2*. The ISGPS risk classification included: type A: 25.9% (*n* = 239), type B: 12.8% (*n* = 118), type C: 25.4% (*n* = 234) and type D: 35.9% (*n* = 331); with corresponding POPF rates of 11.7%, 18.6%, 18.8% and 33.3% respectively (*Table 2*). The ISGPS 3-tier risk classification included type A: 25.9% (*n* = 239), type B: 38.2% (*n* = 352) and type C: 35.9% (*n* = 331), with a corresponding POPF rate of 11.7%, 18.8% and 33.3% respectively. The FRS risk classification included negligible risk: 10.6% (*n* = 98), low risk: 22.2% (*n* = 206), moderate risk: 58.3% (*n* = 537) and high risk: 8.8% (*n* = 81), with respective POPF rates of 11.5%, 13.4%, 25.7% and 34.4%. The a-FRS risk classification included low risk: 22.9% (*n* = 212), intermediate risk: 24.9% (*n* = 229), high risk: 52.2% (*n* = 481), with respective POPF rates of 10.5%, 23.5% and 33.1%. The ua-FRS risk classification included low risk (<5%): 0.5% (*n* = 5), intermediate risk (5–20%): 35.6% (*n* = 328) and high risk (>20%): 63.9% (*n* = 589), with corresponding POPF rates of 0%, 11.7% and 28.2% respectively.

**Table 2:** Distribution of the risk scores and rates of postoperative pancreatic fistula in 919 patients undergoing robotic pancreatoduodenectomy

Type	Distribution	POPF B/C
**ISGPS** *(% of corresponding POPF risk)		
Type A (3.5%): hard texture, pancreatic duct >3 mm	239 (25.9)	28 (11.7)
Type B (6.2%): hard texture, pancreatic duct ≤3 mm	118 (12.8)	22 (18.6)
Type C (16.6%): soft texture, pancreatic duct >3 mm	234 (25.4)	44 (18.8)
Type D (23.2%): soft texture, pancreatic duct ≤3 mm	331 (35.9)	110 (33.2)
**ISGPS 3-tier** *(% of corresponding POPF risk)		
Type A (3.8%): hard texture and pancreatic duct >3 mm	239 (25.9)	28 (11.7)
Type B (14.4%): soft texture or pancreatic duct ≤3 mm	352 (38.2)	66 (18.8)
Type C (29.6%): soft texture and pancreatic duct ≤3 mm	331 (35.9)	110 (33.2)
**FRS** *(% of corresponding POPF risk)		
Negligible risk	98 (10.6)	11 (11.5)
Low risk	206 (22.2)	28 (13.4)
Moderate risk	537 (58.3)	138 (25.7)
High risk	81 (8.8)	28 (34.4)
**a-FRS** *(% of corresponding POPF risk)		
Low risk	212 (22.9)	22 (10.5)
Moderate risk	229 (24.9)	54 (23.5)
High risk	481 (52.2)	159 (33.1)
**ua-FRS** *(% of corresponding POPF risk)		
Low risk (<5%)	5 (0.5)	0 (0)
Intermediate risk (5–20%)	328 (35.6)	38 (11.6)
High risk (>20%)	589 (63.9)	166 (28.2)

Values are *n* (%). *(% of corresponding POPF risk which were originally published). POPF, postoperative pancreatic fistula; ISGPS, International Study Group on Pancreatic Surgery; FRS, fistula risk score; a-FRS, alternative fistula risk score; ua-FRS, updated alternative fistula risk score.

The main characteristics and outcomes of patients with and without POPF grade B/C are displayed in *Table 1*. At baseline, patients with POPF had a higher rate of soft pancreatic texture and smaller main pancreatic duct size. Looking at final pathology, the rate of pancreatic ductal adenocarcinoma was higher in patients without POPF, although the overall rate of malignancy was not significantly different between groups. Patients with POPF had higher rates of major morbidity, postpancreatic haemorrhage grade B/C, bile leakage grade B/C, DGE grade B/C, reoperation on and readmission, compared with the patients without POPF.

### Discrimination

**Fig. 1:**
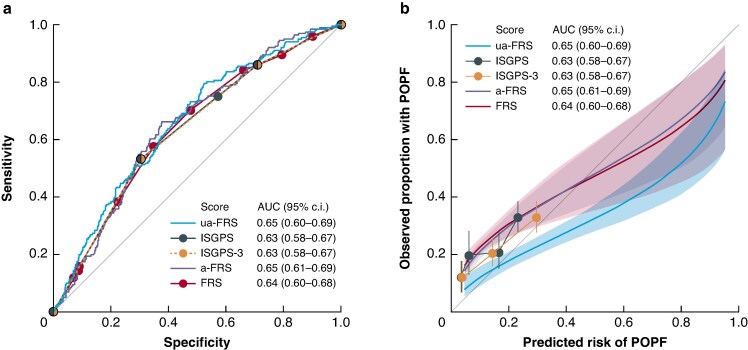
a Discrimination and b calibration of the ISGPS, ISGPS 3-tier and three FRS scores ISGPS, International Study Group on Pancreatic Surgery; POPF, post operative pancreatic fistula; AUC, area under curve; FRS, fistula risk score.

The five FRSs showed moderate discrimination. The AUC of the ISGPS, ISGPS 3-tier, FRS, a-FRS and ua-FRS risk classification, were 0.63 (95% confidence interval (c.i.) 0.58–0.67), 0.63 (95% c.i. 0.58–0.67), 0.64 (95% c.i. 0.60–0.68), 0.65 (95% c.i. 0.60–0.69) and 0.65 (95% c.i. 0.61–0.69); *Fig. 1a*). The ua-FRS did not significantly outperform the ISGPS score (difference in AUC 0.022 (95% c.i. −0.004 to 0.047); *P* = 0.093). In addition, there was no evidence that the ua-FRS outperformed the a-FRS (difference in AUC 0.011 (95% c.i. −0.005 to 0.026); *P* = 0.530) and FRS (0.020 (95% c.i. −0.005 to 0.045); *P* = 0.510). In turn, the ISGPS 3-tier risk score was not outperformed by the ISGPS score (difference in AUC 0.000 (95% c.i. −0.004 to 0.005); *P* = 0.820).

### Calibration

The ISGPS, ISGPS 3-tier, FRS and a-FRS substantially underestimated the risk of POPF, especially in the low-risk categories (*Fig. 1b* and *Table S3*). In patients with ISGPS type A, the observed risk of POPF was higher than the predicted risk of POPF (predicted risk, 3.5%; observed risk, 12% (95% c.i. 8–16); *P* < 0.0001). The risk of POPF was significantly underestimated in patients with ISGPS type B (predicted risk, 6.2%; observed risk, 19% (95% c.i. 13–27); *P* < 0.0001). In contrast, the ua-FRS risk score was well calibrated at low predicted risks, but overestimated the risk of POPF for high-risk patients (*Fig. 1b* and *Table S4*).

### Clinical utility

**Fig. 2:**
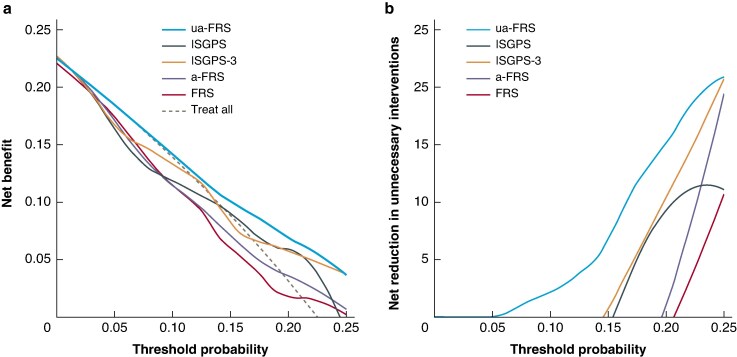
**a Decision curve analysis of the ISGPS, ISGPS 3-tier, and three FRS risk scores, illustrating the net benefit of each risk score**. **b Decision curve analysis of the ISGPS, ISGPS 3-tier and three FRS risk scores, illustrating the net reduction in unnecessary interventions** ISGPS, International Study Group on Pancreatic Surgery; FRS, fistula risk score; a-FRS, alternative fistula risk score; ua-FRS, updated alternative fistula risk score.

In decision curve analysis, the ua-FRS was associated with the highest net benefit compared with the ISGPS, ISGPS 3-tier, FRS and a-FRS across all clinically relevant risk thresholds (0 to 25%). For instance, at a risk threshold of 10%, the ua-FRS score will detect 2 additional patients with POPF per 100 patients compared with the ISGPS score, without increasing the number of false positives (*Fig. 2a*).

In *Fig. 2b*, the net benefit is expressed in terms of the number of unnecessary interventions that are avoided by using the risk scores. At a risk threshold of 10%, the ua-FRS score would be equivalent to reducing the number of unnecessary interventions by 3 per 100 patients, without missing patients with POPF. In contrast, at the same risk threshold, the ISGPS, ISGPS 3-tier, FRS and a-FRS would result in 16, 4, 21 and 21 extra unnecessary interventions per 100 patients respectively.

The discrimination, calibration and net benefit estimates were similar in a sensitivity analysis that included only patients from 2018 to 2021 (*Figs S1* and *S2*).

## Discussion

This international multicentre study demonstrates that nearly two-thirds of patients undergoing RPD have a significant risk of POPF grade B/C. Based on potential clinical utility, the ua-FRS outperforms the ISGPS, ISGPS-3 tier, FRS and a-FRS to predict the risk of POPF although all five scores have moderate predictive value.

The original ISGPS cohort showed a POPF rate of 3.5%, 6.2%, 16.6% and 23.3% for types A, B, C, D^11^  *versus* 11.7%, 18.6%, 18.8% and 33.3% in the present cohort respectively. This again demonstrates the higher rates of POPF in the selected patients undergoing RPD. The 22.2% rate of POPF after RPD in this study is only slightly higher when compared with other studies on RPD^3,22–24^. Most strikingly, a 2021 single-centre retrospective study from the USA reported a POPF rate of only 7.8% after RPD^3^. A clear explanation for this large difference in POPF rates is lacking, although it may be related to the multicentre design of the present study, including different learning curves. Moreover, a lower rate of malignant tumours was found in the present study (58.6% *versus* 82.8%), which may contribute to the higher incidence of POPF.

In 2021, a nationwide study using data from the Dutch Pancreatic Cancer Audit on both open, laparoscopic and robot-assisted pancreatoduodenectomy externally validated the ISGPS risk score^10,11^. The authors reported an AUC of 0.70 (95% c.i. 0.68–0.72), higher than the AUC of 0.63 (95% c.i. 0.58–0.67) found in the present analysis after RPD. The difference may be explained by the different surgical approach, since in the previous study only 20.3% of the patients received MIPD (14.5% RPD and 5.8% laparoscopic pancreatoduodenectomy). Moreover, similar rates of POPF in the ISGPS 4-tier risk score for types B (12.2%) and C (15.6%) were found in the previous study. Therefore, it was suggested that the B and C risk classes may be combined, leading to an ISGPS 3-tier system, which also displayed a more balanced patient distribution^11^. This finding is in line with the present study, where similar rates for type B (18.6%) and type C (18.8%) in the ISGPS 4-tier risk score were found. Finally, the authors reported comparable discrimination for the two ISGPS scores. This was also confirmed in the present analysis, as the AUC was similar for the ISGPS risk score and the ISGPS 3-tier risk score suggesting that the ISGPS 3-tier would be an acceptable alternative to use in patients undergoing RPD.

The ua-FRS was previously validated for open and laparoscopic pancreatoduodenectomy in both Europe and Asia^25–28^. A 2022 prospective study from China, including 400 laparoscopic pancreatoduodenectomies, found an AUC of 0.72 for the ua-FRS^26^. Furthermore, a 2023 prospective study from The Netherlands, including 1358 pancreatoduodenectomies, among which 265 were MIPD, found an AUC of 0.70 (95% c.i. 0.68–0.71)^25^. These AUCs were therefore higher compared with AUCs found in the present study. This may be explained by the different types of surgical approach used in these previous validation studies, although it is unclear by what mechanism the robotic approach influences these findings.

The validation of POPF risk scores in patients undergoing RPD is especially relevant as POPF represents a major concern in RPD^29^. This concern is based on both the high POPF risk in these patients due to patient selection (that is without vascular contact, hence less obstruction and more often a soft pancreas) and the potentially more severe consequences of POPF in RPD (that is number of adhesions, hence higher bleeding risk in patients with POPF)^30–32^. Therefore, it is important to stratify patients in comparative studies of RPD *versus* open pancreatoduodenectomy based on their POPF risk. The present study helps in this respect. Surgeons might use POPF risk scores to counsel patients undergoing RPD and make decisions on patient management (for example drain placement, proactive monitoring, the use of somatostatin analogues and hydrocortisone). However, when using these scores, keep only the moderate AUC and calibration in mind. However, relying solely on the AUC to assess a risk score does not inherently capture a model’s clinical usefulness. Despite a high diagnostic accuracy, poor calibration can significantly impact clinical decision-making. For instance, a model that under- or overestimates the risk of POPF may result in under- or overtreatment. In this study, we found that the ISGPS, ISGPS 3-tier and the ua-FRS demonstrated moderate calibration. Among these, the ua-FRS displayed the highest clinical utility within POPF decision thresholds ranging from 0 to 25%. Decision curve analyses suggest that employing the ua-FRS optimally suits scenarios favouring a low-threshold approach in postoperative care. Consequently, the ua-FRS may be a valuable tool for clinicians in making informed decisions as has been suggested previously^33^. However, it is important to note that the choice for a specific threshold may differ among centres. Nevertheless, the ua-FRS consistently demonstrated better clinical utility within the 0–25% risk range. Thus, even with slight variations in decision thresholds, the ua-FRS can still be used by clinicians who prefer a low decision threshold. The superior predictive capability of the ua-FRS in predicting POPF may be attributed to its continuous nature, contrasting with the categorical nature of the ISGPS and ISGPS 3-tier risk score. Further research is warranted to identify the best model suited for specific clinical and geographical contexts.

The results of this study must be interpreted considering some limitations. First, there were missing values for important variables such as pancreatic texture and pancreatic duct, however, multiple imputation was used to account for these missing variables. Second, the retrospective nature of this study introduces the risk of information bias. Third, the RPD procedures were performed at ten different centres, possibly resulting in heterogeneity. Fourth, the learning curve of each surgeon was not considered in the analysis. This may be especially relevant in RPD, which is characterized by a ‘mastery’ learning curve of 80 procedures in trained centres^34^. Future prospective studies are required to confirm the predictive value and clinical impact of the described scores. Fifth, this study is geographically limited to Europe. A strength of this study is the contribution of four European countries within the E-MIPS consortium, which enhances the generalizability of findings in Europe and reflects a diverse patient population. Moreover, all available FRS risk classifications were taken into account (FRS, a-FRS and the ua-FRS)^7–9^.

## Supplementary Material

zraf036_Supplementary_Data

## Data Availability

The data that support the findings of this study are available from the corresponding author (M.G.B.) upon reasonable request.
