# Supplementary material for: Predicting postoperative pancreatic fistula after robotic pancreatoduodenectomy using International Study Group on Pancreatic Surgery and fistula risk scores: European multicentre retrospective cohort study
Source: BJS Open. 2025 May 7;9(3):zraf036. doi: 10.1093/bjsopen/zraf036 (PMC12056937; doi:10.1093/bjsopen/zraf036)
Supplement: zraf036_Supplementary_Data [file zraf036_supplementary_data.docx]

**Title: Predicting post-operative pancreatic fistula after robotic pancreatoduodenectomy using ISGPS and FRS scores:**

European multicentre retrospective cohort study

**Authors**

Anouk M.L.H. Emmen, MD^1,2,3^; Mahsoem Ali, BSc^3,4^, Bas Groot Koerkamp, MD, PhD^5^, Ugo Boggi, MD^6^, I. Quintus Molenaar, MD, PhD^7^, Olivier R. Busch, MD, PhD ^1,3^, Thilo Hackert, MD, PhD ^8^, Luca Moraldi, MD, PhD ^9^, J. Sven Mieog, MD, PhD ^10^, Daan J. Lips, MD, PhD ^11^, Olivier Saint-Marc, MD, PhD ^12^, Misha DP Luyer, MD, PhD ^13^, Susan van Dieren, MSc^1^, Geert Kazemier, MD, PhD ^3,4^, Felix Nickel, MD^8,14^, Sebastiaan Festen, MD, PhD ^15^, Hjalmar C. van Santvoort, MD, PhD ^7^, Emanuele F. Kauffmann, MD, PhD ^6^, Roeland F. de Wilde, MD, PhD ^5^, Mohammad Abu Hilal, MD, PhD ^2^*, Marc G. Besselink, MD, MSc PhD ^1,3^* for the European Consortium on Minimally Invasive Pancreatic Surgery (E-MIPS)

1 Amsterdam UMC, location University of Amsterdam, Department of Surgery, Amsterdam, the Netherlands

2 Department of General Surgery, Istituto Ospedaliero Fondazione Poliambulanza, Brescia, Italy

3 Cancer Centre Amsterdam, the Netherlands

4 Amsterdam UMC, location Vrije Universiteit, Department of Surgery, Amsterdam, the Netherlands

5 Department of Surgery, Erasmus MC Cancer Institute, Rotterdam, the Netherlands

6 Division of General and Transplant Surgery, University of Pisa, Pisa, Italy

7 Department of Surgery, Regional Academic Cancer Centre Utrecht, St. Antonius Hospital and University Medical Centre, Utrecht, the Netherlands

8 Dept. of General, Visceral and Thoracic Surgery, University Hospital Hamburg-Eppendorf, Hamburg, Germany

9 Department of Oncology and Robotic Surgery, Careggi University Hospital, Florence, Italy

10 Department of Surgery, Leiden Universitair Medisch Centrum, Leiden, the Netherlands

11 Department of Surgery, Medisch Spectrum Twente, Enschede, the Netherlands

12 Service de Chirurgie Digestive, Endocrinienne et Thoracique, Centre Hospitalier Universitaire Orleans, Orleans, France

13 Department of Surgery, Catharina Hospital, Eindhoven, the Netherlands

14 Department of Surgery, University Hospital of Heidelberg, Heidelberg, Germany

15 Department of Surgery, OLVG, Amsterdam, the Netherlands

**Corresponding author.**

Prof. Marc G Besselink, MD MSc PhD

Department of Surgery, Amsterdam UMC

University of Amsterdam, Cancer Centre Amsterdam,

De Boelelaan 1117 (ZH-7F), 1081 HV Amsterdam

Email: [m.g.besselink@amsterdamUMC.nl](mailto:m.g.besselink@amsterdamUMC.nl)

Prof. Mohammad Abu Hilal, MD PhD FRCS

Department of Surgery, Istituto Ospedaliero Fondazione Poliambulanza, Brescia, Italy

Via Leonida Bissolati, 57, 25124 Brescia

E-mail: [abuhilal9@gmail.com](mailto:abuhilal9@gmail.com)

**During review**:

A.M.L.H. Emmen, a.emmen@amsterdamUMC.nl

**Twitter: @AnoukEmmen, @MarcBesselink, @Abuhilal9Abu**

**Supplementary Materials - Index**

| **Supplementary Figures and Tables** |  |
| --- | --- |
| Supplementary table 1. Missing data for all variables. | *Page 3* |
| Supplementary table 2. ISGPS, ISGPS-3 tier and three FRS risk scores including their corresponding POPF risk as originally published. | *Page 4* |
| Supplementary table 3. Calibration of the ISGPS, ISGPS 3-tier, and FRS score. | *Page 5* |
| Supplementary table 4. Calibration of the a-FRS and the ua-FRS score. | *Page 6* |
| Supplementary figure 1: Discrimination and calibration of the fistula risk scores, restricted to patients included in 2018 to 2021. | *Page 7* |
| Supplementary figure 2: Net benefit of the fistula risk scores, restricted to patients included in 2018 to 2021. | *Page 8* |

**Supplementary Tables**

**Supplementary table 1: Missing data for all variables**

| **Variables** | **RPD**  **(n=919)** | |
| --- | --- | --- |
| **Baseline characteristics** | | |
| Age | 1 | (0.1) |
| Sex | 0 | (0) |
| BMI | 70 | (7.6) |
| ASA ≥3 | 130 | (14.1) |
| **Intraoperative outcomes** | | |
| Pancreatic texture | 251 | (27.3) |
| Pancreatic duct size | 95 | (10.3) |
| Operative time | 187 | (20.3) |
| Intra operative blood loss | 220 | (23.9) |
| Conversion | 0 | (0) |
| **Postoperative outcomes** | | |
| Major morbidity (CD≥3) | 0 | (0) |
| Post-operative pancreatic fistula | 3 | (0.3) |
| Post-pancreatectomy haemorrhage grade B/C | 17 | (1.8) |
| Bile leak grade B/C | 13 | (1.4) |
| Delayed gastric emptying grade B/C | 17 | (1.8) |
| Chyle leak grade B/C | 37 | (4.0) |
| Reoperation | 37 | (4.0) |
| Readmission | 37 | (4.0) |
| 30-day/in-hospital mortality | 74 | (8.1) |
| **Oncological outcomes** | | |
| PDAC | 0 | (0) |
| Malignant | 0 | (0) |
| All data are presented as n (%). Variables that are not shown have no missing data.  *ASA = American Society of Anaesthesiologists classification, BMI = body mass index, CD = Clavien-Dindo, PDAC = pancreatic ductal adenocarcinoma, POPF = postoperative pancreatic fistula.* | | |

| **POPF risk score with the % of corresponding POPF risk as originally published** | |
| --- | --- |
| **ISGPS ^9^** | **Risk** |
| Type A: hard texture, pancreatic duct >3mm | 3.5% |
| Type B**:** hard texture, pancreatic duct ≤3mm | 6.2% |
| Type C**:** soft texture, pancreatic duct >3mm | 16.6% |
| Type D**:** soft texture, pancreatic duct ≤3mm | 23.2% |
| **ISGPS 3-tier ^10^** |  |
| Type A**:** hard texture and pancreatic duct >3mm | 3.8% |
| Type B**:** soft texture OR pancreatic duct ≤3mm | 14.4% |
| Type C**:** soft texture and pancreatic duct ≤3mm | 29.6% |
| **FRS ^7^** |  |
| Negligible (0 points) | 0.6% |
| Low risk (1-2 points) | 5.5% |
| Intermediate risk (3-6 points) | 12.9% |
| High risk (7-10 points) | 24.2% |
| **a-FRS, ua-FRS ^17,8^** |  |
| Low risk | <5% |
| Intermediate risk | 5-20% |
| High-risk | >20% |

**Supplementary table 2. ISGPS, ISGPS-3 tier and three FRS risk score including their corresponding POPF risk as originally published.**

**Supplementary table 3: Calibration of the ISGPS, ISGPS 3-tier and FRS score**

| **Type** | **Predicted risk of POPF** | | **Observed risk of POPF (95% CI)** | | **P value** |
| --- | --- | --- | --- | --- | --- |
| **ISGPS** |  |  |  |  |  |
| Type A | 3.5% |  | 12% | (8-16%) | **<0.0001** |
| Type B | 6.2% |  | 19% | (13-27%) | **<0.0001** |
| Type C | 16.6% |  | 19% | (14-24%) | 0.38 |
| Type D | 23.2% |  | 33% | (28-38%) | **<0.0001** |
| **ISGPS 3-tier** |  |  |  |  |  |
| Type A | 3.8% |  | 12% | (8-16%) | **<0.0001** |
| Type B | 14.4% |  | 19% | (15- 23%) | **0.023** |
| Type C | 29.6% |  | 33% | (28-38%) | 0.15 |
| **FRS** |  |  |  |  |  |
| Negligible risk | 0.6% |  | 12% | (5-23%) | **0.017** |
| Low risk | 5.5% |  | 13% | (8-21%) | **0.015** |
| Intermediate risk | 12.9% |  | 26% | (22-30%) | **<0.0001** |
| High risk | 24.2% |  | 34% | (25-46%) | **0.037** |
| The P value is for a test that compares the observed risk of POPF to the predicted risk of POPF; i.e., P<0.05 indicates that the risk of POPF is significantly over- or underestimated. | | | | | |

**Supplementary table 4: Calibration of the a-FRS and the ua-FRS score**

**a-FRS**

| **Performance measure** | **Estimate (95% CI)** | | **P value** | |
| --- | --- | --- | --- | --- |
|  |  |  |  | |
| Intercept | 0.72 | (0.54 to 0.90) | **<0.0001*** | |
| Slope | 0.55 | (0.33 to 0.77) | * | |
| Observed/expected ratio | 1.71 | (1.50 to 1.95) | **<0.0001** | |
| *: recalibration test (2 degrees of freedom) for the null hypothesis that intercept=0 and slope=1. P<0.05 indicates that the intercept and/or slope are significantly different from their ideal values | | | |  |

**Ua-FRS**

| **Performance measure** | **Estimate (95% CI)** | | **P value** | |
| --- | --- | --- | --- | --- |
|  |  |  |  | |
| Intercept | –0.46 | (–0.63 to –0.29) | **<0.0001*** | |
| Slope | 0.56 | (0.37 to 0.74) | * | |
| Observed/expected ratio | 0.75 | (0.66 to 0.84) | **<0.0001** | |
| *: recalibration test (2 degrees of freedom) for the null hypothesis that intercept=0 and slope=1. P<0.05 indicates that the intercept and/or slope are significantly different from their ideal values | | | |  |

**Supplementary figure 1: Discrimination and calibration of the fistula risk scores, restricted to patients included in 2018 to 2021**

**
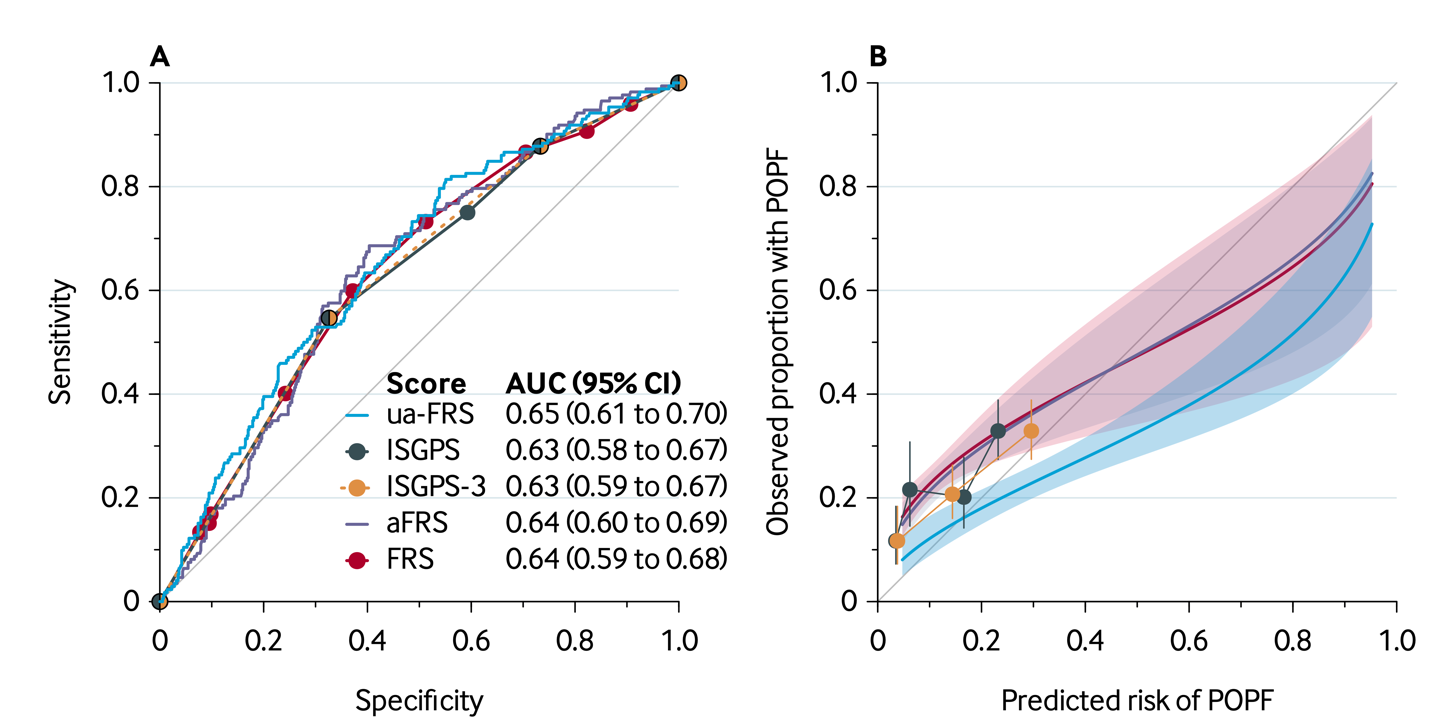
**

**Supplementary figure 2: Net benefit of the fistula risk scores, restricted to patients included in 2018 to 2021**

**
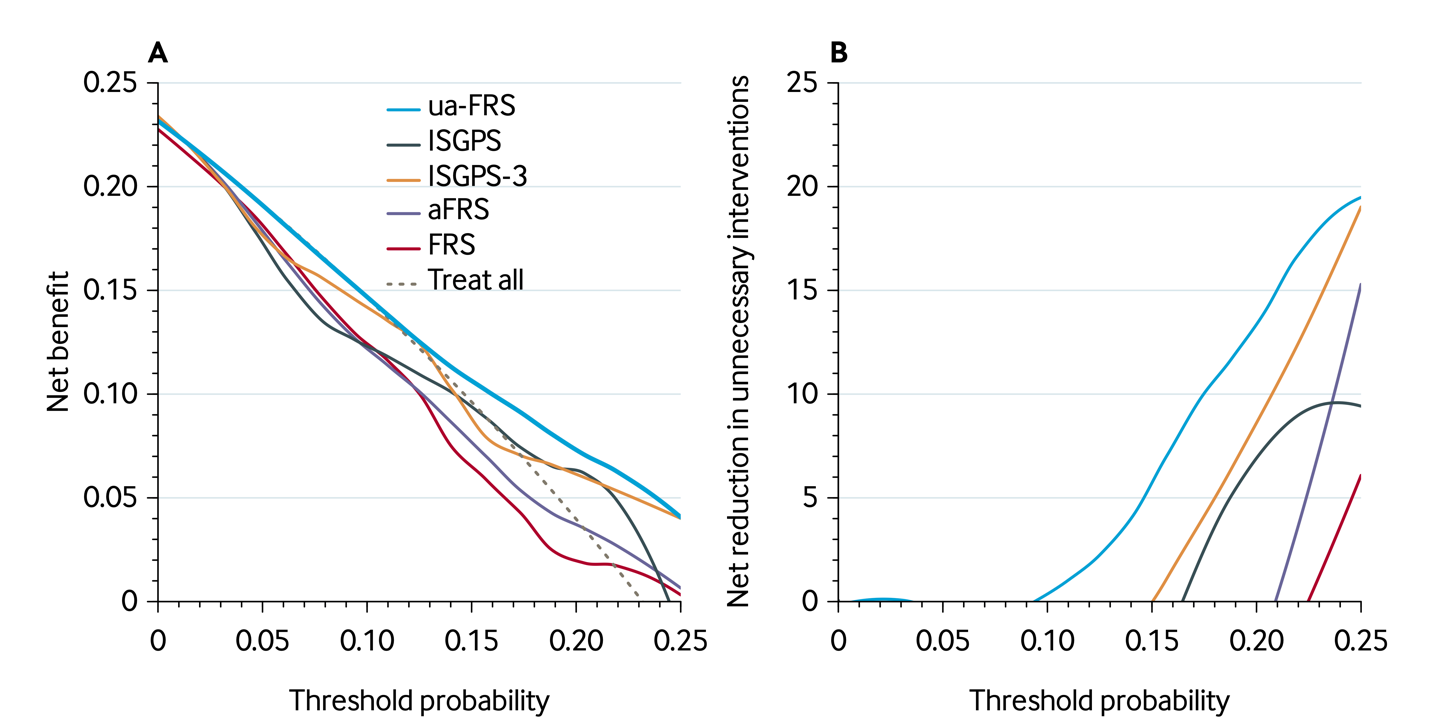
**
